# Editorial: Advances in livestock genetics: enhancing breeding practices and improving animal health

**DOI:** 10.3389/fgene.2026.1932330

**Published:** 2026-07-27

**Authors:** C. S. Wilson, A. S. M. Cesar, F. M. Rezende

**Affiliations:** 1 USDA-ARS-Range Sheep Production Efficiency Research Unit, Dubois, ID, United States; 2 University of São Paulo, Piracicaba, Brazil; 3 University of Florida, Gainesville, FL, United States

**Keywords:** animal breeding, animal health, breed of origin, genetics, genomics, transcriptome wide association studies, underlying biological traits

## Introduction

As research in the fields of livestock genetics and genomics moves forward, new technologies and traits of interest have emerged that are motivated by the goals of improving animal productivity, enhancing animal health, and gaining a greater understanding of functional traits. Our definition of the livestock industry continues to expand beyond traditional species, as this Research Topic demonstrates, including research ranging from meat rabbits to “insects as food and feed”.

The 15 manuscripts in this Research Topic cover eight species and a multitude of modern and visionary technologies to better understand traits of interest, including the Breed-of-Origin (BOA) effects in beef cattle and transcriptome-wide association studies (TWAS) in rabbits.

## RNA technology

Further understanding of athletic performance in horses was provided by Su et al. in Yili horses and by Gong et al. in Mongolian horses. Su et al. evaluated blood collected before and after a race to assess exercise-induced changes in gene expression. The authors evaluated mRNAs, lncRNAs, and circRNAs to advance our understanding of physiological adaptation to exercise. Gong et al. evaluated the molecular basis by which ferulic acid, a natural antioxidant, regulates muscle fiber type transformation. Differentially expressed miRNAs and lncRNAs were identified in skeletal muscle cells, providing further understanding of their potential impact on athletic performance and muscle function. In their review article, Liu et al. provided an in-depth description of how lncRNAs affect gene expression and various biological processes, particularly the impact of myogenesis on muscle development and lipogenesis on fat deposition.

## Animal health

Diseases affecting livestock are costly and a concern for animal wellbeing. Ahmad et al. reported that outbreaks of Infectious Pancreatic Necrosis Virus (IPNV) in rainbow trout stocks can result in 10%–100% mortality. Through an IPNV challenge study, the research team concluded that genomic selection should be used to improve resistance to IPNV in rainbow trout. In Tanzanian dairy cattle herds, animal diseases are estimated to cause mortality rates of up to 14%. Hernandez-Castro et al. evaluated antibody responses to zoonotic and reproductive infectious diseases in Tanzanian dairy cattle and estimated genetic parameters for several health traits, with heritability estimates ranging from 0.03 to 0.44. The authors used genome-wide association studies (GWAS) to identify loci associated with seropositivity to several diseases and pathogens, providing a basis for selecting genetic resilience to animal diseases.

## Genetic diversity and robustness

Two studies of genetic diversity in sheep furthered our understanding of local adaptation to the environment. Tian et al. evaluated the genetic structure and diversity of seven Tibetan sheep breeds to understand how these sheep have uniquely adapted to their harsh environment. Similarly, Wang et al. evaluated the genetic diversity of Teha sheep, a cross between the Texel and Kazakh breeds, for the first time. The researchers identified critical genomic regions that contribute to the breed’s adaptability. In Brazilian buffalo populations, Santana et al. addressed concerns related to inbreeding depression and decreased genetic variability. To address these challenges, the authors evaluated the PRDM9 gene, which promotes the formation of crossing-over points across the genome. This strategy can be used to increase the frequency of specific alleles, generate new haplotypes, and increase genetic variability.

For some species, selection occurs at the group level rather than the individual level; however, the potential loss of genetic diversity in the population remains a concern. As part of the “insects as food and feed” industry, black soldier flies are selected as a group, making the loss of genetic diversity and the development of sustainable breeding programs a challenge. Dufresne et al. used genotype-by-sequencing technology to assign parentage with fewer than 200 SNPs, enabling pedigree-based selection.

Two manuscripts focused on understanding robustness in livestock species. Terenina et al. assessed the genetic determinism of hypothalamic-pituitary-adrenocortical (HPA) activity and its influence on production and robustness traits in pigs. Harari et al. investigated the functional longevity of show-jumping horses due to concerns related to animal wellbeing and injuries. As with longevity traits across species, there is a need to identify early indicators of longevity rather than waiting until the animal is mature. To evaluate functional longevity earlier in life, these authors evaluated blood parameters at rest in young horses (2–4 years old) and identified 16 parameters that may be predictive of their genetic value for functional longevity, allowing for earlier evaluation of this trait.

## Novel traits and technologies

As we collect vast amounts of high-throughput phenotypic data on animals, we need strategies to evaluate and interpret these large quantities of data and to identify economically important phenotypes. To address this issue, in a series of two manuscripts, Anas et al. first evaluated beef cattle heifers for underlying biological traits (UBTs), which are a set of interrelated traits. The researchers used factor analysis and Bayesian network learning to better understand traits associated with reproduction, body conformation, and carcass measures. In their second manuscript, the authors used GWAS to further evaluate the UBT and found that the heritability estimates for the UBT were higher than those for the individual traits. The need for heat tolerance in beef cattle in warm climates has made *Bos taurus x Bos indicus* crossbreeding a common solution. In order to understand the genetic architecture of heat tolerance traits, Zayas et al. first determined the Breed-of-Origin (BOA) for each SNP marker. Then, BOA, SNP, and BOA + SNP models were evaluated to identify QTL associated with thermotolerance. The authors recommended that BOA + SNP models be used in other studies in which more than one ancestral breed is being evaluated.

Finally, using a novel technology for livestock species, a transcriptome-wide association study (TWAS) was used in meat rabbits to identify candidate genes whose mRNA expression levels may be involved in regulating carcass and meat traits. He et al. identified limitations associated with GWAS and the ability of TWAS to overcome said limitations. Their study provides an example of how TWAS can be used by researchers in future studies.

## Summary

As our capacity for genetic and genomic evaluation continues to expand, it is important to remain focused on understanding traits of economic and biological importance and translating this knowledge into strategies that promote genetic improvement while enhancing animal wellbeing and health. The manuscripts included in this Research Topic further our understanding of trait function through RNA technology, genomic selection for improved animal health, methods to understand and increase genetic diversity, and strategies to group traits so that we can begin to understand the vast amount of data that we are collecting.

This Research Topic highlights the growing importance of methodological advances integrating genetics, genomics, and systems biology to address major challenges facing current livestock production. As the global demand for animal-derived foods continues to increase alongside concerns related to animal health, environmental sustainability, climate resilience, and food security, innovative breeding strategies have become essential. The studies gathered in this Research Topic demonstrate how advanced genomic technologies and functional analyses can accelerate genetic improvement, support precision livestock farming, and enhance the development of healthier, more resilient, and more resource-efficient production systems. This is an exciting time to be involved in livestock genetics and genomics research, and we hope this Research Topic inspires researchers to explore novel methods and improve breeding practices and animal health. Collectively, these contributions provide valuable scientific knowledge while strengthening the bridge between fundamental research and practical applications for the future of sustainable animal agriculture.

